# Supplementary material for: Clinical progression and outcomes of patients hospitalized with COVID-19 in humanitarian settings: A prospective cohort study in South Sudan and Eastern Democratic Republic of the Congo
Source: PLOS Glob Public Health. 2022 Oct 19;2(10):e0000924. doi: 10.1371/journal.pgph.0000924 (PMC10021555; doi:10.1371/journal.pgph.0000924)
Supplement: S1 Table — (DOCX) [file pgph.0000924.s001.docx]

**Supplemental Table 1.** Characteristics of Health Facilities Enrolling Cases in this Study

|  | **Democratic Republic of the Congo** | | | **South Sudan** |
| --- | --- | --- | --- | --- |
| **Hospital Location, Type and Study Enrollments** | | | | |
| Location | Bukavu,  South Kivu | Goma,  North Kivu | Goma,  North Kivu | Juba,  Central Equatoria |
| Sector | Public | Public | NGO operated | NGO operated |
| Study patients admitted at facility | 25 | 42 | 22 | 55 |
| **Hospital Capacity** | | | | |
| Doctors | 86 | 36 | 42 | 9 |
| Nurses | 168 | 92 | 86 | 33 |
| COVID care beds / Total beds^1^ | 22/380 | 20 / 220 | 28/220 | 82/82 |
| Staff with COVID-19 training^2^ | No | Yes | Yes | Yes |
| PPE - face masks and gloves^3^ | Available | Available | Available | Available |
| Power supply | Always | Always | Always | Often |
| **Critical Care Oxygen Supply^4^** |  |  |  |  |
| Oxygen concentrators + cylinders | Adequate supply | Adequate supply | Limited supply | Adequate supply |
| Flowmeters for oxygen therapy | Available | Not available | Limited supply | Available |
| Oxygen delivery interfaces | Available (reused) | Not available | Available (reused) | Available (reused) |
| BIPAP & CPAP Machines | Available | Not available | Available | Available |
| Mechanical Ventilators | Not available | Not available | Not available | Available |
| **Diagnostic/Monitoring capacity** |  |  |  |  |
| Pulse oximeter | Available | Available | Available | Available |
| Electrocardiogram | Available | Available | Available | Not available |
| Chest x-ray | Available | Available | Available | Not available |
| CT scan | Available | Not Available | Not available | Not available |
| **Laboratory Capacity^5^** |  |  |  |  |
| Basic Metabolic Panel | Available | Available | Available | Not available |
| Inflammatory markers | Available | Available | Available | Not available |
| **Critical Medications** |  |  |  |  |
| Steroids | Available | Available | Available | Available |
| Remdesivir^6^ | Not Available | Not Available | Available | Available |
| Vasopressors | Available | Available | Available | Available |
| Antibodies or convalescent plasma | Not available | Not available | Not Available | Not Available |
| ^1^The SSD facility was a COVID-19 only referral hospital; ^2^ Staff considered trained if they received information on COVID-19 case management and infection prevention and control (IPC) for COVID-19 prior to study initiation; ^3^Including single use surgical masks and KN94/KN95 masks and disposable latex gloves; reusable eye protection was available in all facilities except the private hospital in Goma; ^4^BIPAP/CPAP machines and ventilators were available in some facilities, a number of factors limited the use of invasive oxygen therapies including irregular electricity, lack of supplies and human resource constraints; ^5^Basic metabolic panel includes complete blood panel, liver and kidney function; inflammatory markers including c-reactive protein, ferritin and/or sedimentation rate (ESR); ^6^In DRC, remdesivir was a second line treatment (first line = hydroxychloroquine) and less severe cases may not have met eligibility protocol. | | | | |
