# Supplementary material for: Clinical progression and outcomes of patients hospitalized with COVID-19 in humanitarian settings: A prospective cohort study in South Sudan and Eastern Democratic Republic of the Congo
Source: PLOS Glob Public Health. 2022 Oct 19;2(10):e0000924. doi: 10.1371/journal.pgph.0000924 (PMC10021555; doi:10.1371/journal.pgph.0000924)
Supplement: Attachment — Submitted filename: R1 Response to Comments 3Mar22.docx [file pgph.0000924.s003.docx]

**PGPH-D-21-01027 – Response to Reviewer Comments (Revision 1)**
Clinical Progression and Outcomes of Patients Hospitalized with COVID-19 in Humanitarian Settings: A Prospective Cohort Study in South Sudan and Eastern Democratic Republic of the Congo

**Journal Requirements**

1. Please include a complete copy of PLOS’ questionnaire on inclusivity in global research in your revised manuscript. Completed. African research team members that led the research in each country (Abdou Sebushishe and Mbong Eta Ngole) are listed as authors and additional local team members are included either as members of the research team or in the acknowledgements section.

2. Please provide separate figure files in .tif or .eps format only and remove any figures embedded in your manuscript file. Please ensure that all files are under our size limit of 20MB. Figures have been removed from the manuscript and provided in the requested file format.

3. In the online submission form, you indicated that your data will be submitted to a repository upon acceptance. We strongly recommend all authors deposit their data before acceptance, as the process can be lengthy and hold up publication timelines. Data has been uploaded in Humanitarian Data Exchange and is ready for release (by the authors). Data will be made public once the manuscript has been accepted.

4. Please amend your detailed Financial Disclosure statement. This is published with the article, therefore should be completed in full sentences and contain the exact wording you wish to be published. A financial disclosure statement was added to the title page document that reads as follows: *This study was funded by the United States Agency for International Development (award 72OFDA20GR0221, Principal Investigator: Doocy). The funders had no role in study design, data collection and analysis, decision to publish, or preparation of the manuscript.*

**Reviewer 1**

Introduction

- Why the authors highlighted the remdesevir treatment in the introduction section given that there is no specific treatment against COVID-19? Text has been revised to remove remdesivir.

Methods

- The authors did not clearly define the inclusion and exclusion criteria for the study Inclusion and exclusion criteria have been added to the methods along with COVID-19 case definitions and testing information. The authors did not clearly define the COVID-19 case and how the diagnostic was made? On base of serological test? Antigenic test? Or RT-PCR? Inclusion and exclusion criteria have been added to the methods along with COVID-19 case definitions and testing information.
- The authors must specify how the sampling of the study was done? Has a sample size been calculated? Sampling has been clarified – all eligible participants consenting to participate were enrolled. Sample size calculations were conducted based on the primary study aims (risk factor analysis); this has been clarified in revisions to the text and is also cited; discussion on sample size calculations for this paper has also been added to the methods section.
- Operational definition must be clearly presented in the method section. Operational definitions are included in Table 2 which is now referenced in the methods section.

Results

- Unlike many studies, fever was not a major symptom in this study. How the authors can explain it? Text has been added to the results section to include self-reported fever, which was more frequent than observed fever, and to note that fever was found less often than anticipated. In the discussion, text and a citation have also been added noting that other studies of hospitalized patients have observed low fever rates, that fever is dynamic and a smaller proportion of patients will have fever at certain point in time compared to ever over the course of COVID-19 illness, and that self-medication with antipiretics is common and could have affected the reported fever rate.
- How the authors can explain a high proportion of lack of laboratory test in the prospective study context? Lack of availability of laboratory testing was a major challenge as noted in the limitations section (and discussed in more depth in one of the companion papers). This was an observational study and testing capacity in both countries (and the African continent generally) is known to be limited, thereby contributing to under-reporting of cases; there was also testing hesitancy in both locations. We have add in the limitations lack of funding for tests – as the study did not include budget for clinical management of cases.
- We did not add more detail in the manuscript in regard to this point due to length considerations and the volume of comments received.
- The authors reported in results section that only one patient in DRC received vasopressor treatment but this information doesn’t appear in table 3. This information is presented in supplemental Figure 1; because only a single patient received this class of medication odds of survival (table 3) could not be calculated.
- Can the authors provide a staging of patient by OMS COVID-19 classification? We are not able to provide staging per the WHO definition because not all of the required data points were available (most notably chest imaging by x-ray or CT).
- Can the authors provide an operational definition for acutely ill? How the authors can explain the fact that some acutely ill patients were treated ambulatory? This was an observational classification made by clinicians based on their own observations/judgement at enrollment; acutely ill were classified into ambulatory and non-ambulatory to provide further clarification on perceived severity.
- In the table 3, the authors must specify for hypotension and hypertension the variable systolic blood pressure (SBP) and diastolic blood pressure (DBP) We assumed this comment for Table 2 and have made this correction.
- Which other antiviral was used in DRC COVID-19 patients? (table 3) Lopinavir and Ritonavir were the other antivirals used; this has been added as a footnote in the table.

Discussion

- The mortality rate reported in this study is very low compared to other study specially in DRC. The authors must discuss this discrepancy. Discussion and references have been added.
- The authors have justified the high mortality in SSD by the later presentation and worse conditions at admission of SSD patients. Can the authors specify if the criteria of admission in the medical center was the same in DRC and Sudan? There were not set study admission criteria in country due to the observational nature of the research, however, in both locations facilities should have been following available MoH guidance when screening and admitting patients.
- Only 18.8% of patients have presented fever. Can the authors discuss this results? This is the same as the point raised in the results section, where we have responded as follows: Text has been added to the results section to include self-reported fever, which was more frequent than observed fever, and to note that fever was found less often than anticipated. In the discussion, text and a citation have also been added noting that other studies of hospitalized patients have observed low fever rates, that fever is dynamic and a smaller proportion of patients will have fever at certain point in time compared to ever over the course of COVID-19 illness, and that self-medication with antipiretics is common and could have affected the reported fever rate.
- The authors can add a DRC studies on COVID-19 especially in the ACCCO study, no study from DRC or Sudan was included. Here two studies from DRC: Nlandu, Y., Mafuta, D., Sakaji, J. et al. Predictors of mortality in COVID-19 patients at Kinshasa Medical Center and a survival analysis: a retrospective cohort study. BMC Infect Dis 21, 1272 (2021). https://doi.org/10.1186/s12879-021-06984-x; Makulo, JR., Mandina, M.N., Mbala, P.K. et al. SARS-CoV2 infection in symptomatic patients: interest of serological tests and predictors of mortality: experience of DR Congo. BMC Infect Dis 22, 21 (2022). <https://doi.org/10.1186/s12879-021-07003-9> Thank you for providing these references. We have incorporated them into the first paragraph of the discussion to help contextualize findings related to overall mortality rate, as well as the finding that time from onset to hospitalization is associated with mortality.

**Reviewer #2**

Thank you for this work. I think it is really important and I am glad to see this experience is being documented. I am sorry if the comments are many but if there are multiple papers coming from this work, I thought it might be worth discussing some of the issues in more detail, if possible. I see the strength of this paper as documenting COVID in populations where little has been documented. We need greater insight into clinical presentations and severity in different settings. I would be cautious on drawing conclusions on clinical severity risk factors or the effectiveness of interventions from a small dataset, as the epidemic has been impacted so much correlations that did not prove as meaningful with further analysis. I think it’s really important the paper discusses underweight status. Being overweight has been such a clear risk factor, but in other settings low weight is so often correlated with other factors (say cancer) that it is hard to tease out. I am really glad to see this discussed as a factor as 2 years into the epidemic it has not had the attention and analysis it should.

Abstract
Can the Findings abstract be more specific?
"The observed mortality proportion among hospitalized patients was 16.7% (CI: 11.2-23.3%) and was greater in South Sudan, where patients presented later after symptom onset and in worse conditions."
Maybe list specific mortality or say, rate is over 3x higher in South Sudan Interpretation. We revised to note the mortality rate is 3x higher in SSD.

Page 4
Endnote error in first line. We were unable to locate this error.

Was pregnancy assessed? This is certainly a risk factor that causes a substantial loss of life years. Non-pregnant women often have a better survival than men but pregnant women have worse outcomes and in this context would presume would be a frequent enough risk factor.
<https://jamanetwork.com/journals/jamapediatrics/fullarticle/2779182>

Pregnancy could not be analyzed due to small sample size (only one participant was pregnant).

Seroprevalences. it would be good to cite existing data on these two contexts. serotracker does not include good studies on either area but there are more recent studies.

https://pubmed.ncbi.nlm.nih.gov/34013872/

https://www.ajtmh.org/view/journals/tpmd/104/4/article-p1526.xml

https://www.panafrican-med-journal.com/content/article/38/93/full/
This would be important to document for future readers in a world where past infection is common and immunity based on past infections and vaccines will shape a different global epidemic.

We have added the citations above and now provide seroprevalence estimates for both Eastern DRC and Juba SSD in the introduction.

Remdesivir (page 4) is commonly used in the US but is not recommended in many countries because of perceived lack of utility. The WHO maintains a conditional recommendation against use of remdesivir. <https://www.who.int/publications/i/item/WHO-2019-nCoV-therapeutics-2022.1>
I might just stress that evidence based, targeted therapeutics were not available. When this paper will be read, monoclonal antibodies and Paxlovid will be the drugs for which access inequities will be the concerns. There are many in the WHO and other organizations and countries that do not feel the impact of remdesivir is significant enough (there’s a lot of different studies with different marginal findings). We have revised the introduction and discussion to not point specifically to remdesivir and we now reference antivirals/monoclonal antibodies and evidenced-based treatments more generally.

Page 5
Duration of symptoms before presentation: were there any expected delays related to the SSD center being a COVID referral center? Were there extra hurdles to presentation because of this? (This is a common issue in outbreaks where infectious diseases are treated outside of the main hospital system). Were there any other reasons documented why there were delays? (I see later the discussion on denial and other issues, anything more that can document this would help). It would be interesting to have insight into this as this may play a substantial role in affecting mortality.

In the discussion we note that higher mortality in SSD was likely attributable to delays in case presentation and patients presenting at the facility in worse condition. We did not specifically enquire about delays in relation to facility type, thus we cannot provided a definitive answer on the reason for delayed care. We are aware that testing hesitancy and difficulty attaining tests and test results was a major reason for delayed care in both settings. It is definitely possible that patients referred from other facilities could have faced longer transfers delays in SSD, however, this is unlikely to have impacted this study because nearly all study participants presented directly at facilities or were referred by mobile teams [not other hospitals].

Page 6
Is it possible to quantify oxygen use? Concentrators vs tanks? Amount of total oxygen that can be delivered? COVID requires high quantities of oxygen and in low resource settings this is often the real limitation. It’s hypoxia (oxygen) and not ventilation (help breathe) that is the main barrier. As such, CPAP is not used that much for COVID even when available and IPC concerns don’t limit use. Rather high flow (like say 60L/min) of oxygen is preferred, even when ventilators and cpap is available. This can be created with oxygen tanks in low resource settings (stringing together multiple tanks). Concentrator machines cannot provide the same amount of oxygen that tanks can; maybe at most 5L/min and of lower percentages of oxygen (80-90%, rather than 98% or more from a tank that reads 5 L/min)
How many tanks of oxygen available per person? What oxygen use rates?
In low resource settings where this very expensive resource is so often limited, there is often a tendency to conserve to avoid running out so patients aren’t able to reach the levels we would have rathered.
Is there monitoring of oxygen and standardized protocols for use? Are oxygen levels kept above certain levels? Are there thresholds at which futility is reached?
Oxygen in low resourced settings is quite expensive, in part because of resource limitations but also monopolies, <https://www.yahoo.com/now/lack-oxygen-leaves-patients-africa-080052014.html>
and especially in South Sudan this is a major limitation to care. First plant came online in Sept. <https://www.afdb.org/en/news-and-events/press-releases/south-sudan-countrys-first-oxygen-plant-comes-stream-juba-hospital-help-fight-covid-19-45393>
Concentrator machines were often used for COVID in South Sudan and this would be a major limitation on the extent of care that can be delivered.

Available equipment (e.g. ventilators, BIPAP/CPAP, oxygen concentrators is shown for each facility in Supplemental Table 1. SSD had the best availability, and equipment type and availability varied in DRC. Unfortunately amount of oxygen used was not a data point we collected in the study so we cannot address this point and instead have noted this as a limitation.

Do we know anything about deaths that occurred without oxygen being given? Those who die largely require oxygen so would clarify if had any other info on those deaths with no oxygen. Was it available? Were there lapses in monitoring? There can be some cases of sudden clots but in general almost everyone, where there is close monitoring and available resources, would need oxygen.

There were 24 patients that died and of these 23 received oxygen while hospitalized. The remaining case was an 80 year old woman who had an O2 of 94, respiratory distress and mild pneumonia at admission.

Is it known if steroids were given before patients needed oxygen? Steroids have been found to benefit those who have severe COVID but are considered a risk for those without severe COVID or not needing oxygen. The WHO has a “conditional recommendation against systemic corticosteroids in patients with non-severe COVID-19” The risk is of worsened diabetes, secondary infections, and all the side effects of steroids without the benefit. In many low resource settings, steroids have been given out to those who do not need oxygen (O2 is not <90 or not <94, there are different cut offs) in order to do something, but may be causing more harm than good.
<https://www.who.int/publications/i/item/WHO-2019-nCoV-therapeutics-2022.1> <https://www.thelancet.com/article/S2213-2600(20)30530-0/fulltext>

Unfortunately, temporality of the various treatments received was not analyzed, largely due to the frequency of data collection and the short time period that inpatients who became deceased were in the study; we have added this as a study limitation.

Also, vasopressors given without close monitoring are more dangerous than helpful. They aren’t normally lifesaving interventions without greater clinical capacity.
Because hypoxia alone is the major driver of severe disease along with clots, vasopressors are not as crucial here as in say cases of bacterial sepsis. They may help with bacterial superinfections or in patients who are intubated and sedated, but they are only adjunctive treatments to bridge a patient.

Vasopressors were only received by several patients in the study.

Page 7
Is there any quantification of how many patients arrived near death? Total death figures? Arrival at or near death is of course a common and under-reported factor affecting the reporting of mortality of many outbreaks and so anything to quantify it or total death rates could help.

In DRC, 24 people died before study enrollment (2.3% of the DRC sample); in South Sudan this information was not collected. We have added this detail in the beginning of the discussion where mortality rates are addressed.

Is there another paper besides #17 that can be cited as the manuscript is not available? It’s an important topic.

Unfortunately there is no alternate citation, however, the paper is in press and the citation can be updated/ finalized prior to publication of this manuscript.

It is really important that paper shows a finding on underweight. Since the beginning of the epidemic, this has been a question with few answers or focus. This is great to see, even though the number (8) enrolled was small. I might stress the need to study this further.

We agree the further investigation of underweight is of interest from a personal perspective, but given that there was no significant association between underweight and mortality it is difficult to justify adding this to the conclusions/recommendations.

I would edit and qualify further the statement about co-infections. It’s not just that the numbers are low, the diagnostic accuracy is also low. Given the low rates of testing for malaria, it is hard to make a conclusion about malaria, especially as treatment for malaria did not correspond to RDT+ tests. Moreover, as malaria infection can correlate with age and other risk factors like pregnancy, it would need a larger dataset to make any clear conclusions, even if we knew precisely who had malaria and who did not. (If interested, this article collected a number of different studies on malaria and COVID though I think they jumped to some conclusions without clear basis: <https://bmcinfectdis.biomedcentral.com/articles/10.1186/s12879-022-07064-4> ) The rates of reported past TB are quite low relative to what would be expected (ie if 1 in 300 develop the disease in a year a past history of TB of 1.1% is quite low). So I am not sure we can feel confident that we have a good grasp on the effects of these diseases. There is a very robust literature on HIV and COVID. There are many who do quite well with COVID who have HIV, even though overall HIV increases risk, so I would not expect to necessarily see an effect in this sample size.

We have noted concerns under-reporting at the end of the section on laboratory test results in the results section; we also note issues with self-reporting of prior medical conditions and malaria testing in the limitations section.

I would cite further sources if discussing risk of anemia in COVID. Some have found it to increase mortality. Others have seen the opposite, higher red blood cell counts, to be associated with mortality (disease does cause clotting). It likely also is often a correlative factor and say a marker for other risks (men/testosterone, pregnancy, sickle cell, cancer etc) so can easily vary in different populations.
<https://bmjopen.bmj.com/content/11/2/e044618.long>

Citation #23 refers to a meta-analysis that shows anemia increased risk for poor COVID-19 outcomes; interested readers can see a number of recent studies and their findings summarized in this source.

Please edit the statement "Vitamin C and multivitamins provision to inpatients however
pointed to a protective effect (marginally significant), which is consistent with several studies,28,29 though evidence to date is inconclusive.30,31" Vitamin C was not significant here (nor was it with MVI) in the adjusted Odds table and I would be very careful with trends or values bordering significance. Any statements on therapies that have been studied and routinely shown not to work should be carefully worded. There are many reasons why people receiving one therapy may improve without a directly causal relationship; there may be other factors (like attention to detail or ability to swallow extra meds, which may not be captured in this study). Vitamin C and multivitamins have after many studies not been shown to be effective at this time. There is certainly a difference in vitamin C in different populations, like as Mija Ververs has shown in young male refugees from South Sudan and certainly it would be interesting to see if provision does make a difference, but this would be a different study. However, study after study has shown that they have not been effective. These studies have been cherrypicked by those who do not believe in vaccines to offer alternatives like cocktails of ivermectin, HCQ, and vitamin C, allowing patients to blame doctors and nurses and not those advocated against vaccination. These faux treatments have also been sent to those with fewer resources who do not have access to medical care. Hence I’m always nervous when seeing Vit C described as marginally significant. In fact, papers cited as 28 and 29 actually are studies which have shown that vitamin C has not been effective. Source 29 states as conclusion: "We did not find significantly better outcomes in the group who were treated with HDIVC in addition to the main treatment regimen at discharge." Source 28 states "This pilot trial showed that HDIVC failed to improve IMVFD28, but might show a potential signal of benefit in oxygenation for critically ill patients with COVID-19 improving PaO2/FiO2 even though.”

We have revised the included citations to incorporate another review/meta-analysis of Vitamin C that was recently published and edited the text to indicate Vitamin C provision is not associated with improved COVID-19 outcomes.

Human resources is discussed. Was there any impact of when someone presented? Any impact of the strike on mortality? Were there clinical surges where mortality or other outcomes was affected? Was there any difference in mortality over time?

Human resource themes are discussed in depth in one of the forthcoming companion papers (citation 16); the citation will be added prior to publication of this manuscript (the paper is provisionally accepted). The strike in SSD was among MoH workers and did no impact the participating facility; we did not assess mortality trends over time due to the concern that not all facility deaths were included in our sample which could lead to inaccurate conclusions.

Looking back on this epidemic, it will be important to clarify vaccination rates (as it may not be clear to future readers how non-existent vaccination was well into 2021) and the impact of variants and past infection (seroprevalence) on outcomes. Mortality will be different in different phases of the epidemic as we continue on, given differences in variants and immunity. So it would help readers in the future if documenting any info on surges and variants and specifics on vaccine inequity. It appears generally that in March 2021 there was a surge in South Sudan, while Jan and July 2021 surges in DRC and Rwanda. There is some documentation on variants found, but not a lot of course in these contexts, so certainly do not need to find what cannot be found, but may make mention of changing variants worldwide.
<https://radiotamazuj.org/en/news/article/covid-19-south-sudan-witnesses-resurgence-confirms-delta-variant>
To address this concern, we added limited availability of vaccines in the introduction and updated the seroprevalence citations. The surges are discussed in the companion paper (citation 16).

Table 1
Can you provide info on the size of the catchment area? Distance for travel? North and South Kivu are obviously much larger than Juba. What populations or geographic areas do these clinics cover?

We attempted to include this information in earlier versions of the table, but decided we could not do so with confidence and thus elected to exclude the information. The facilities were generally perceived as mostly receiving patients from within the same health zone, but a challenge in comparing population sizes covered is that the participating hospitals were one of several in an urban area. If considered critical, we can include this information in the table however our preference is not to add this because the figures would be gross estimates and based on many questionable assumptions.

Would change “Clinical staff trained” to “Staff had COVID training” or something similar. Thee footnote may not be read and I don’t want readers thinking there are untrained doctors and nurses. The row label has been revised to address this comment.
Are all the nurses and doctors specifically RNs and MDs or are there also nursing assistants and CO’s or other providers? Was there a dedicated number of providers from the mixed use facilities who worked with COVID patients. There were also lower level staff, these counts were more variable based on staffing structure and not included in the table; the health facility assessment did not ascertain if providers were shared across wards, and given that this may have changed over time we did not add this as a row in the table.

Might not say N95 equivalents, but just say surgical masks, KN94, or KN95 or what was available. Is there a sense of how many had surgical masks and how many N95s? Just better for future documentation to understand. The footnote has been revised to address this comment.

Did patients have continuous pulse oximetry? Any info on how regularly pulse ox was checked?
Is there any info on antibiotics used and how frequently? Data in Table 1 is from a health facility assessment. Availability of devices was assessed but regularity of use among COVID-19 patients was not. Pulse ox was checked as part of study observations, but due to the observational nature of the study, there was no effort to systematize use in routine care. Only COVID-19 critical medications were reported in Table 2, which excludes antibiotics, because the table is intended to summarize COVID-19 management capacity across sites.

Table 2
Those who did not have symptoms but were admitted, any idea what admission for? Were these admitted for non-COVID diagnoses? Health appearance was based on an initial visual observation by providers and is not equivalent to asymptomatic.

Would stress that Self-reported prior tuberculosis (despite heading) as these numbers are lower than expected. Some studies use CXR criteria for prior TB so it’s good to add self-reported. The self-reporting of medical history is noted in both the methods and limitations.

Definition of ARDS has an “OR” where there should be an “AND” It should also be 3 and not 2
ARDS defined as acute onset (≤1 week of new/worsening respiratory distress) AND PaO2/FiO2 ≤300
mmHg OR SpO2/FiO2 ratio ≤315 (adults) or ≤264 (children); AND pulmonary edema not explained by fluid overload or cardiac failure; OR bilateral opacities on chest X-ray/CT scan not explained by effusions, lung collapse or nodule; Make ARDS 3 and then change the other numbers so the footnotes line up. There are two footnotes labelled as 2 (SIRS and ARDS) and this means the footnotes don’t line up. We have made the AND/OR correction to the ARDS footnote and corrected the numbering mistake.

Figure 3 shows a very high use of antibiotics. Only 3-7% of COVID cases on admit are found to have coinfections with bacteria. There is certainly a lot of overuse of antibiotics which, if not benefiting pt, is exposing them to harm (diarrhea, AMR, renal/hepatic/allergic/cardiac side effects). If possible, I would explore antibiotic use more. Is there any access in the higher resource clinics to micro testing? Microbiology lab? (clearly not in the lower resourced SSD clinic but perhaps in the DRC clinic with a CT scanner). Bacterial superinfections occur much more commonly with intubation which unfortunately was not a possibility here. Unfortunately, laboratory testing was very limited and not systematically done in the participating facilities [as would be anticipated in higher-resource settings], as such the requested information is not available for analysis. High use of antibiotics is likely a result of treatment protocols (e.g. the DRC COVID-19 treatment protocol indicates all patients should receive Azithromycin).

**Reviewer #3:**

The manuscript is technically sound and uses sound data to support the conclusions. However, there are some typographical errors:
- Under the introduction: the last line on paragraph 2; the use of the word 'concerns' should be revised. I would suggest it is replaced with 'inadequate'. The suggested revision has been incorporated.

- Under the methods: the second last line on paragraph 1, I suggest that the sentence extract, '.....a secondary aim was to document clinical progression and characterise COVID-19 management' is rewritten. This sentence extract should be rewritten as '...a secondary aim was to document clinical progression and characterise COVID-19 clinical management'. The suggested revision has been incorporated.

Under the results: I suggest that the sentence, 'While the mortality proportion differed significantly between SSD and DRC overall, there was no difference in survival probability when adjusted for from the date of symptom onset', be rewritten. The sentence should be rewritten as, 'While the mortality proportion differed significantly between SSD and DRC overall, there was no difference in survival probability when adjusted for the date of symptom onset'. The suggested revision has been incorporated.

**General remarks**

The paper presented clinical profile of and outcomes of admitted COVID-19 patients in five health facilities in the Democratic Republic of Congo and South Sudan – both in sub-Saharan Africa. As reported elsewhere, the study showed COVID-19 survival is influenced by presence of underlying illnesses like diabetes as well as timely access to quality care right from the time the symptoms of illness commence. These findings are therefore critical and justify the need to prop up COVID-19 surveillance and case management to avert adverse outcomes and improve survival. The manuscript is technically sound and uses sound data to support the conclusions.

**Specific comments and suggestions**

- Please state how the sample size was determined for the study. Alternatively, if these were the total number of patients that could be enrolled, it is critical that a power calculation is done ensure that the study was adequately powered. If the power determination reveals the study was not adequately powered, then the limitations should be updated accordingly. Sample size calculations were conducted based on the primary study aim of characterizing risk factors for poor COVID-19 outcomes (presented as a separate paper). Because this is a secondary aim and descriptive (i.e. we were not looking to detect significant differences between countries/facilities or test a hypothesis) we did not conduct power calculations.
- It is important that the authors state the case definition for COVID-19 cases that were included in the study. This is critical for assessing the role of misclassification bias in the study. The case definition has been added.
- Similarly, the authors should provide the definitions for the outcomes (COVID-19 recovery and COVID-19 death) used in the study. Clear definitions and objective determination of the outcomes are critical for minimizing the risk of bias in the assessment of the outcome. The methods section indicates that recovered is defined as discharged from hospital care; death was the other outcome but a definition did not seem necessary since cause was not considered.
- Similarly, the admission criteria for patients should be stated. It is also possible that these criteria differed in the two countries where the study was undertaken given the country-specific capacities for managing COVID-19 patients. How were these county and pandemic phase specific changes and differences in admission criteria for facility based care adjusted for in the study? Inclusion and exclusion criteria were added. Models were adjusted for a variety of parameters including country, as a fixed effect and facility as a random effect.
- Besides malaria and self-reported diabetes, please state how the presence of other underlying diseases like HIV, tuberculosis, and other non-communicable diseases were assessed among cases enrolled into the study? Due to the observational nature of the study, patient history (e.g. HIV, TB, diabetes) were self-reported; in the case of diabetes patients, HbA1C was measured. Diagnostic test results were recorded for study purposes if ordered by a physician, however, in the case of malaria many suspect cases were not tested. This theme is discussed in the methods, results and limitations.
